# Assessing the Effects of Farm Management Systems and Diarrhea on Gut Microbiota and Metabolites in Dairy Calves in Indonesia

**DOI:** 10.3390/ani16121766

**Published:** 2026-06-08

**Authors:** Andi Hiroyuki, Jahidul Islam, Ainissya Fitri, Rusli Fidriyanto, Ki Ageng Sarwono, Andhika Yudha Prawira, Edy Sophian, Rohmatussolihat, Wulansih Dwi Astuti, Sukarman, Delicia Yunita Rahman, Yantyati Widyastuti, Natsuki Ohtani, Ryota Hirakawa, Mutsumi Furukawa, Roni Ridwan, Tomonori Nochi

**Affiliations:** 1 International Education and Research Center for Food and Agricultural Immunology, Graduate School of Agricultural Science, Tohoku University, Sendai 980-8572, Japan; andi.hiroyuki.q5@dc.tohoku.ac.jp (A.H.);; 2 Laboratory of Animal Functional Morphology, Graduate School of Agricultural Science, Tohoku University, Sendai 980-8572, Japan; 3 Laboratory of Animal Mucosal Immunology, Graduate School of Agricultural Science, Tohoku University, Sendai 980-8572, Japan; 4 Laboratory of Animal Data Science & Innovation, Graduate School of Agricultural Science, Tohoku University, Sendai 980-8572, Japan; 5 Research Center for Applied Zoology, National Research and Innovation Agency (BRIN), Cibinong 16911, West Java, Indonesia; aini002@brin.go.id (A.F.); rusl006@brin.go.id (R.F.); kiag001@brin.go.id (K.A.S.);; 6 Research Center for Animal Husbandry, National Research and Innovation Agency (BRIN), Cibinong 16911, West Java, Indonesia; yant001@brin.go.id; 7 Division of Mucosal Vaccines, International Vaccine Design Center, The Institute of Medical Science, The University of Tokyo, Tokyo 108-8639, Japan; 8 Department of Animal Biosciences, University of Guelph, Guelph, ON N1G 2W1, Canada; 9 School of Nutrition and Health Sciences, Taipei Medical University, Taipei City 11031, Taiwan; 10 Tohoku Center for Teaching and Learning, Institute for Excellence in Higher Education, Tohoku University, Sendai 980-8576, Japan

**Keywords:** microbiota, metabolites, calves, smallholder, commercial dairy system

## Abstract

Dairy production in Indonesia relies on two contrasting calf-rearing systems: concentrate-driven commercial dairy systems (CDS) and smallholder farms (SH). As these systems differ in feeding practices, they may influence gut development in calves. This study examined how farm management affects the fecal microbiota and metabolites in healthy and diarrhea post-weaning calves. Fecal samples were collected from 11 and 14 calves from CDS and SH facilities, respectively. Gut bacterial communities were analyzed using 16S rRNA gene amplicon sequencing, and metabolites were characterized using untargeted gas chromatography–mass spectrometry (GC-MS). Microbial diversity, metabolite profiles, and correlation networks were compared between the farm types and diarrhea status. Our results showed that farm management strongly shaped microbial community structure and metabolic outputs, but did not alter microbial richness. Although diarrhea is typically associated with reduced microbial richness, it did not affect the microbial community structure, suggesting that it primarily impacted microbial function, particularly the metabolic environment. Correlation network analysis revealed stronger linkages between microbes and metabolites in SH calves, especially under healthy conditions. Overall, these findings indicate that dietary structure is a key determinant of fermentation stability, with CDS calves showing greater metabolic instability. In contrast, SH calves maintain a more resilient, fiber-driven functional state.

## Simple Summary

Calves in Indonesia are raised in two very different farming systems, which are concentrate-driven commercial dairy systems and smallholder farms. Commercial dairy farms typically feed post-weaning calves with concentrated feed, while smallholder farms rely more on natural, fiber-rich feeds. Because early diet strongly influences gut development, we aimed to understand how these two management systems shape the gut microbiota and metabolites. We also examined whether diarrhea affected these differences. Fecal samples were collected from post-weaning calves raised in both systems, and both microbial communities and associated metabolites were analyzed. Our findings revealed a strong influence of farm management systems on microbial community structure and metabolic outputs, but not on microbial richness, in developing post-weaning calves. Furthermore, the diarrheal status did not appear to affect the microbial community structure, suggesting that it primarily affected microbial function, particularly the metabolic environment. Pearson’s correlation study revealed that smallholder farm calves exhibited stronger linkages between microbes and metabolites, especially under healthy conditions. These results suggest that dairy management plays a key role in shaping microbe–metabolite interactions in post-weaning calves in Indonesia.

## 1. Introduction

The early-life gut microbial composition of dairy calves is a key determinant of immune development, health, and long-term productivity. The microbial community rapidly develops from birth through the pre-weaning to weaning transition (~90 days), a period highly sensitive to dietary inputs, such as colostrum intake and subsequent feeding strategies [1,2,3]. Early microbial succession during this window is strongly influenced by both maternal factors and environmental exposures, which together shape the establishment of core bacterial taxa essential for gastrointestinal maturation [4,5]. In Indonesia, dairy farming systems can be broadly classified into two types: smallholder (SH) farms, in which post-weaning calves are typically fed whole milk and forage, and large-scale commercial dairy systems (CDSs), in which post-weaning calves are fed with milk replacer and starter feed. Variations in these management practices might contribute to inconsistent calf performance [6,7]. Such differences in feeding strategies, hygiene standards, and housing conditions may further modulate microbial colonization patterns, potentially leading to divergent metabolic and immune developmental trajectories between production systems [8,9,10,11]. However, integrated analyses linking gut microbiota and metabolite dynamics during this critical window remain limited.

In addition to shaping microbial composition, diet regulates metabolic activity by supplying substrates for bioactive metabolites, including volatile fatty acids and tricarboxylic acid cycle intermediates, which are essential for gut development and host metabolism [12]. These metabolites not only serve as energy sources but also act as signaling molecules that influence epithelial proliferation, mucosal barrier function, and rumen papillae development [13,14,15]. Early-life microbial colonization also plays a critical role in gut immune development programming, as commensal bacteria stimulate epithelial maturation, promote gut-associated lymphoid tissue (GALT) formation, and modulate innate and adaptive immune responses [16,17]. Disruptions in this early microbial and immune crosstalk have been associated with heightened inflammatory responses and increased vulnerability to enteric pathogens [18]. These microbially derived signals contribute to the establishment of immune tolerance, enhancement of barrier integrity, and reduced susceptibility to enteric pathogens during the vulnerable weaning period [19]. However, complex interactions among dietary, microbial, environmental, and management factors may disrupt gut microbial balance and metabolic homeostasis, causing gut dysbiosis and diarrheal disease in post-weaning calves, even in well-managed systems [20,21]. Diarrhea during this stage can further disrupt microbial community stability, alter metabolite production, and impair nutrient absorption, thereby creating a detrimental feedback loop that exacerbates growth retardation and health challenges [18,22,23].

In this study, to address this gap, we integrated microbiota and metabolite analyses to compare calves from SH and CDS farms, representing typical production systems in Indonesia. The differences between calves with and without diarrhea during the first 90 days of life were also examined. By focusing on the weaning management, this study provides insights into diet–microbiota–metabolite interactions under healthy and diarrheal conditions, with implications for improving calf health and dairy productivity in Indonesia and other South Asian countries.

## 2. Materials and Methods

### 2.1. Animal Studies

This study was approved by the ethics committees of the National Research and Innovation Agency for investigating gut microbiota in dairy calves (271/KE.02/SK/12/2024). A total of 25 pre-weaning dairy calves were enrolled, consisting of 11 calves from one CDS farm and 14 calves from five SH farms. The number of calves sampled from each system reflected natural differences in herd size and calf availability during routine farm visits. Because of these inherent differences in herd structure, the final sample distribution between CDS and SH farms was unbalanced. Calves were randomly selected from the available post-weaning population at each farm. Fecal consistency was assessed using the scoring system described by Islam et al., which classifies fecal texture on a standardized scale [24]. Fecal scores were defined as follows: scores of 1 (normal) and 2 (soft) were classified as healthy, whereas scores of 3 (loose) and 4 (watery) were classified as diarrhea. In this study, fecal score was used solely as an indicator of intestinal transit and luminal changes rather than as a clinical diagnosis of diarrhea.

### 2.2. Fecal Bacterial DNA Extraction, 16S rRNA Sequencing, and Taxonomic Profiling

Fecal samples were collected directly from the rectum using sterile gloves and immediately transferred into Norgen Stool Nucleic Acid Collection and Preservation Tubes. Total bacterial DNA was extracted from fecal samples and purified using the Norgen Stool DNA Isolation kit (Norgen Biotek, Thorold, ON, Canada) according to the manufacturer’s protocol. The V3–V4 hypervariable region of the bacterial 16S rRNA gene was amplified according to a previous study [25]. All PCR amplicons were sequenced on the Illumina MiSeq platform using the MiSeq Reagent Kit v2 from Illumina Inc., San Diego, CA, USA (500-cycle configuration) [25]. Following next-generation sequencing, the demultiplexed raw reads were retrieved from the Illumina BaseSpace Sequence Hub. Sequence processing and downstream analyses were performed using Quantitative Insights Into Microbial Ecology 2 (QIIME 2) (version 2024.5) [26,27]. After processing data by using the Divisive Amplicon Denoising Algorithm 2 (DADA2), the feature table contained 4551 ASVs and a total of 451,189 high-quality sequences. Per-sample sequencing depth ranged from 5226 to 27,700 reads (median 17,094; mean 18,047). These values indicate that sequencing depth was sufficient for robust microbial diversity analyses. The relative abundance (%) was determined using an operational taxonomic unit (OTU) table with taxonomic annotations generated by QIIME2. The associated metadata file was analyzed using MicrobiomeAnalyst 3.0 [28]. To minimize potential analytical bias, all downstream statistical tests were performed using non-parametric methods (Kruskal–Wallis, PERMANOVA, Linear Discriminant Analysis Effect Size [LEfSe]), which are robust to unequal group sizes. Marginal or borderline results were interpreted cautiously to avoid overgeneralization.

### 2.3. Fecal Metabolites Analysis

The amounts of metabolites in fecal samples were measured as previously described, with some modifications [29]. Briefly, fecal samples were freeze-dried, and metabolites were extracted with a solvent mixture (methanol:chloroform:ultrapure water = 5:2:2) containing ribitol as an internal standard. The supernatant was concentrated under vacuum. Derivatization was performed by adding methoxyamine hydrochloride to pyridine, followed by *N*-methyl-*N*-(trimethylsilyl)trifluoroacetamide (MSTFA). Gas Chromatography–Mass Spectrometry (GC–MS) analysis was performed using a GC–MS–QP2010 Ultra system (manufactured by Shimadzu corporation, Kyoto, Japan) equipped with an RTX-5 MS column, with helium as the carrier gas. Volatile Fatty Acid (VFA) in fecal samples was analyzed using a GC–MS–QP2010 SE system (Shimadzu) equipped with an AOC-20i+s autosampler and a MEGA WAX MS capillary column using ultrahigh-purity helium (99.99%) as the carrier gas. For sample preparation, fecal extracts were mixed with 5% sulfosalicylic acid dihydrate, and a particle-free aliquot was injected at a split ratio of 50:1. VFAs were identified and quantified using a Volatile Free Acid Mix standard (Supelco, St. Louis, MO, USA, CRM46975) [30].

Metabolomic data quality control and preprocessing were performed using MetaboAnalyst 6.0. Missing values were examined across samples, and no features exceeded the missing-value threshold; therefore, no data filtering was applied (Table S6). Data were normalized by sum to account for differences in sample concentration, cube root transformed to reduce the influence of large values and improve normality, and auto-scaled (mean-centered and divided by the standard deviation of each variable) prior to statistical analysis. Differential metabolites were identified using univariate analysis (*t*-test/ANOVA) with false discovery rate (FDR) correction (*q* < 0.05) and a fold-change threshold of |FC| ≥ 1.5. Only metabolites meeting both criteria were considered significant.

### 2.4. Statistical Analysis

Statistical significance was assessed using GraphPad Prism version 9.5.1 (GraphPad Software, San Diego, CA, USA), with a threshold of *p*  <  0.05. Differences between the two groups were evaluated using an unpaired *t*-test. Linear discriminant analysis of effect size was performed using MicrobiomeAnalyst 3.0 [28]. Metabolite analysis was performed using MetaboAnalyst 6.0 [31]. Pairwise Spearman correlations were calculated in R using the cor function, and statistical significance was assessed using the cor.test. *p*-values were adjusted for multiple comparisons using the Benjamini–Hochberg method via *p*.adjust. Correlation patterns were visualized using the pheatmap package in R.

## 3. Results

### 3.1. Characteristics of Calves from CDS and SH Farms

**Figure 1:**
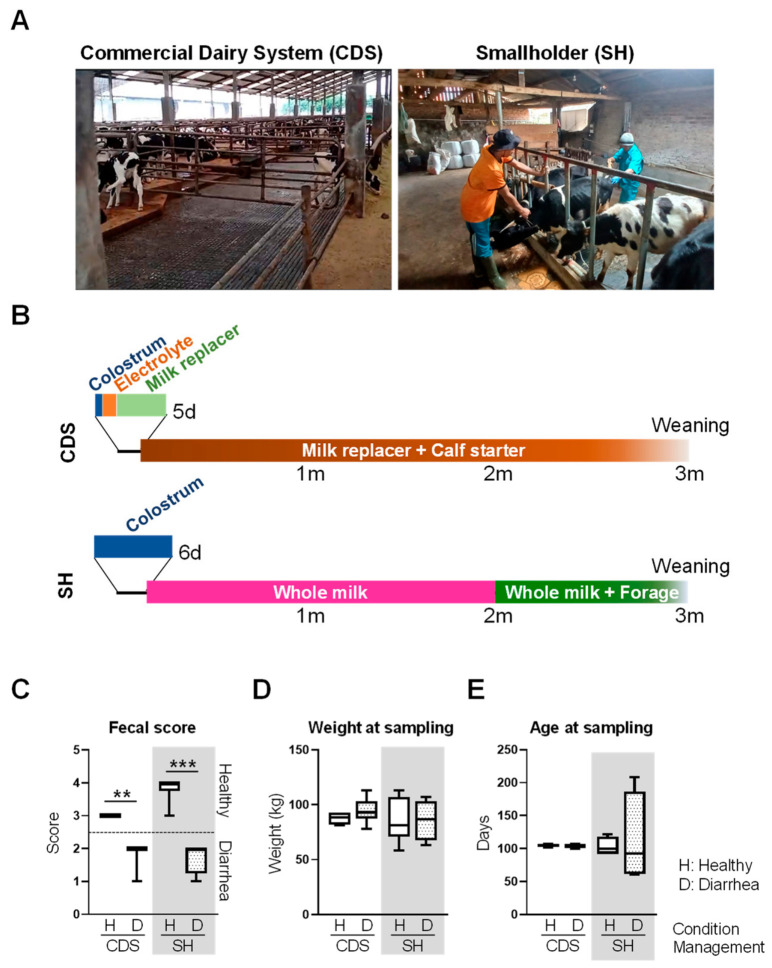
Experimental design of this study. (**A**) Selection of calves from large-scale commercial dairy systems (CDS) and smallholder (SH) in Indonesia. (**B**) Management styles in the CDS and SH systems. (**C**) Fecal scores, (**D**) body weights, and (**E**) ages of calves randomly selected from the CDS and SH groups. Statistical analysis was performed using the Mann–Whitney U test. ** *p* < 0.01, *** *p* < 0.001.

Fecal samples were collected from 11 calves on one CDS farm and from 14 calves across five SH farms in Indonesia (Figure 1A). Figure 1A provides an overview of the sampling structure across the two farm management systems. The main difference between the two systems lies in feeding practices. In the CDS group, calves were provided with milk replacer and calf starter after colostrum feeding during the weaning period, whereas in the SH farms, calves were fed whole milk during the first month after colostrum intake and subsequently received forage alongside continued milk feeding (Figure 1B). Based on fecal scores, four calves were classified as healthy and seven as diarrheal in the CDS group. In contrast, in the SH group, 10 calves were classified as healthy, whereas four were classified as diarrheal (Figure 1C). Therefore, samples were divided into four groups: CDS-Healthy (CDS-H), CDS-Diarrhea (CDS-D), SH-Healthy (SH-H), and SH-Diarrhea (SH-D), to further investigate the effects of calf management systems and diarrhea on microbiota and metabolite profiles. Notably, there were no differences in age or sampling timing after birth among the groups (Figure 1D,E).

### 3.2. Farm Management System Associated with Diarrhea Prevalence and Shapes Calf Gut Microbiota

**Figure 2:**
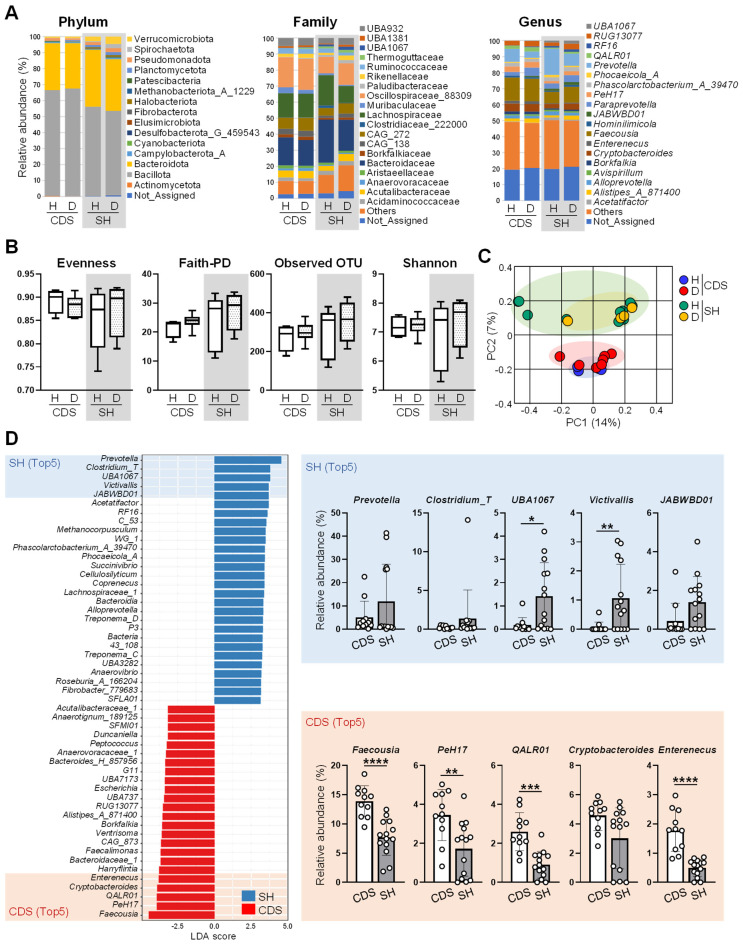
Microbial composition in the CDS and SH groups. Calves on commercial dairy system (CDS) and smallholder (SH) farms were classified by health status as follows: CDS-H (*n* = 4), CDS-D (*n* = 7), SH-H (*n* = 10), and SH-D (*n* = 4). (**A**) Microbial composition at the phylum, family, and genus levels. (**B**) Alpha diversity indices, including evenness, Faith’s phylogenetic diversity (Faith’s PD), observed OTUs, and Shannon index. (**C**) Principal coordinate analysis based on unweighted distance matrices of 16S rRNA gene sequencing data. (**D**) Linear discriminant analysis scores of differentially abundant taxa between the CDS and SH groups. The relative abundances of the top five taxa identified in the CDS and SH groups were compared. Statistical analysis was performed using the Mann–Whitney U test. * *p* < 0.05, ** *p* < 0.01, *** *p* < 0.001, **** *p* < 0.0001.

To investigate differences in the microbiota between the groups, genomic DNA was extracted from 25 fecal samples and subjected to 16S rRNA sequencing after amplification of the V3–V4 regions. Across all post-weaning calves, the fecal microbiota predominantly comprised the phyla *Bacillota* (formerly *Firmicutes*) and *Bacteroidota* (Figure 2A, Supplementary Tables S1–S3). *Bacillota* represented 67.81 ± 10.5% in CDS-H, 67.39 ± 3.73% in CDS-D, 56.20 ± 8.47% in SH-H, and 53.57 ± 5.95% in SH-D, reflecting the typical dominance of Firmicutes-related taxa in young ruminants (Figure 2A, Supplementary Table S1). *Bacteroidota* was the second most abundant phylum, accounting for 27.93 ± 9.58% in CDS-H, 28.31 ± 2.70% in CDS-D, 34.80 ± 11.01% in SH-H, and 32.04 ± 4.85% in SH-D (Figure 2A, Supplementary Table S1). *Bacteroidota* is commonly associated with fiber-degrading activity, which is consistent with its higher proportion in forage-fed calves. At the family level, *Bacteroidaceae* was predominant, with relative abundances of 16.55 ± 11.41% (CDS-H), 16.05 ± 3.13% (CDS-D), 25.44 ± 16.75% (SH-H), and 19.17 ± 10.46% (SH-D) (Figure 2A, Supplementary Table S2). As *Bacteroidaceae* is typically enriched in calves that consume plant-derived substrates, this pattern explains its higher abundance in forage-fed SH groups. An example of a family that was more abundant in the CDS group than in the SH group was *Oscillospiraceae_8399,* with relative abundances of 19.33 ± 3.57% (CDS-H), 19.03 ± 3.03% (CDS-D), 10.36 ± 4.62% (SH-H), and 13.49 ± 2.29% (Figure 2A, Supplementary Table S2). *Oscillospiraceae_8399* acts as a fermenter, digesting the abundant starch found in the guts of the CDS group. At the genus level, *Faecousia* was found to be the most abundant in the CDS group regardless of the diarrhea status (14.68 ± 2.00% in CDS-H and 13.92 ± 3.05% in CDS-D), suggesting that this genus may be associated with calf starter feeding (Figure 2A, Supplementary Table S3).

To investigate overall differences between healthy and diarrheal post-weaning calves in the CDS and SH groups, alpha and beta diversity analyses were conducted. Alpha diversity metrics, including Evenness, Faith’s PD, observed OTUs, and Shannon indices, revealed no differences between healthy and diarrheal post-weaning calves within or across both groups (Figure 2B). In contrast, beta diversity based on unweighted UniFrac revealed a clear difference between the CDS and SH groups (Figure 2C), whereas the diarrhea status did not affect the microbiota within each group. These results indicate that farm management, rather than diarrhea status, primarily shapes the microbial community. To identify the significant microbial taxa in either the CDS or SH group, Linear Discriminant Analysis Effect Size (LEfSe) was conducted by simply comparing the CDS and SH groups irrespective of the diarrhea status (Figure 2D). Taxa with a Linear Discriminant Analysis (LDA) score > 2.5 were identified as key microbial biomarkers for each feeding system. The top five differential genera in the CDS group were *Faecousia*, *PeH17*, *QALR01*, *Cryptobacteroides*, and *Enterenecus*, which are often associated with concentrate-rich or starch-fermenting environments. In contrast, *Prevotella*, *Clostridium_T*, *UBA1067*, *Victivallis*, and *JABWBD01* were the top five differential genera in the SH group and are commonly associated with fiber degradation and plant-derived substrates. These biomarker profiles highlight distinct microbial signatures shaped by farm management, underscoring their role in structuring the post-weaning calf gut microbiota.

### 3.3. Both the Farm Management System and Diarrhea Status Influence the Gut Metabolites

**Figure 3:**
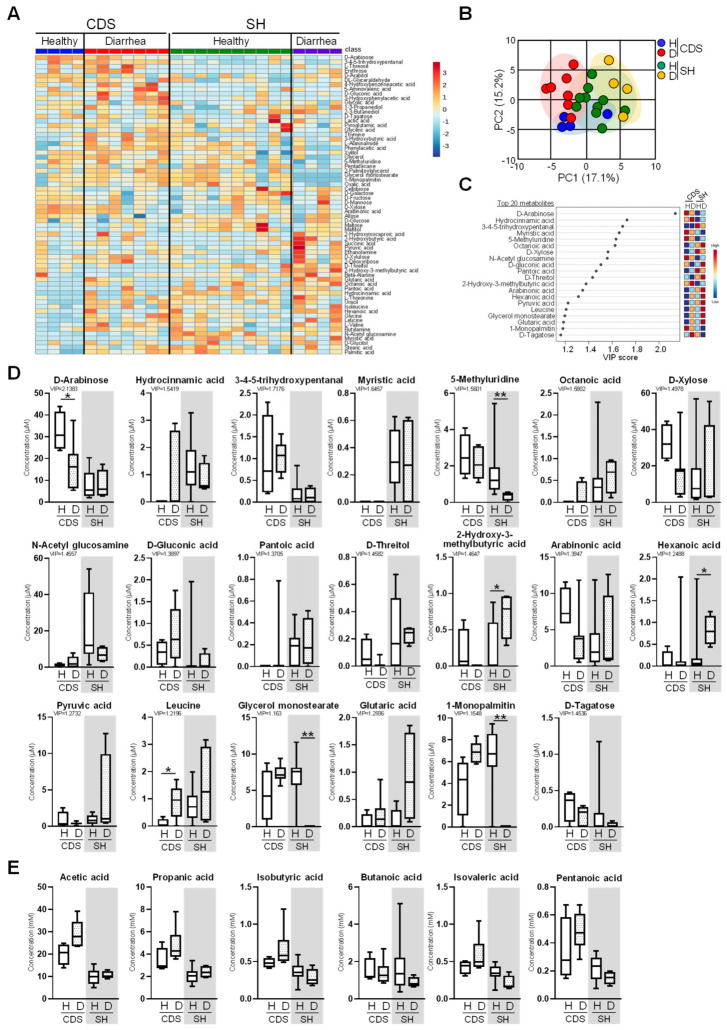
Comparison of microbial metabolite composition in the CDS and SH groups. (**A**) A total of sixty-six metabolites were identified in fecal samples from the CDS-H, CDS-D, SH-H, and SH-D groups by GC-MS. (**B**) Principal component analysis score plot of all groups. (**C**) Metabolites were ranked by contribution (top 20) and presented as variable importance in projection (VIP) scores from partial least-squares-discriminant analysis (PLS-DA). (**D**) The concentration of the top 20 metabolites (**E**) and six volatile fatty acids were compared among the groups. Statistical analysis was performed using the Mann–Whitney U test. * *p* < 0.05, ** *p* < 0.01.

To characterize metabolic differences between the two systems, fecal metabolite profiles from 25 post-weaning calves were compared. A total of 66 metabolites were detected in all samples (Figure 3A, Supplementary Table S4). Ten metabolites showed significant differences between the CDS and SH groups, independent of diarrhea status (Supplementary Table S4). Overall metabolite patterns also differed between the four farm–diarrhea status combinations, indicating that both management systems and diarrhea status influenced fecal metabolite composition (Figure 3B). Multivariate analysis by using Partial Least-Squares-Discriminant Analysis identified the top 20 metabolites that contributed most strongly to group separation (Figure 3C), including higher D-arabinose in CDS-H calves and lower 5-methyluridine in SH-D calves (Figure 3D, Supplementary Table S4). The volatile fatty acid profiles also varied between the systems. Except for butanoic acid, all VFAs were significantly higher in the CDS group (Figure 3E, Supplementary Table S5), suggesting more active saccharolytic fermentation or reduced intestinal absorption during diarrhea. These patterns indicate that calves in the CDS may produce more abundant VFAs due to the rapid supply of fermentable carbohydrate, while elevated fecal VFAs in diarrheic CDS calves may also reflect reduced absorption or increased intestinal transit.

### 3.4. Microbiota–Metabolite Correlation Analysis Across the Farm Management System and Diarrhea Status

**Figure 4:**
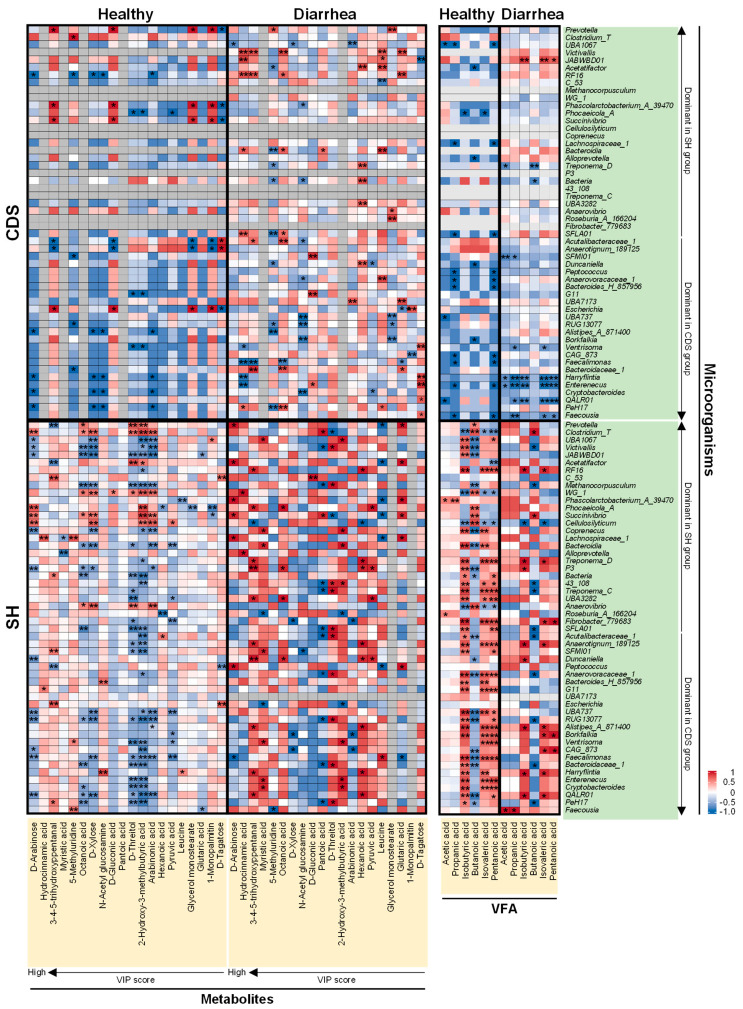
Microbiota–metabolite correlation in the CDS-H, CDS-D, SH-H, and SH-D groups. Pearson’s correlation coefficients were calculated to assess the relationships between 52 keystone microbial taxa and the top 20 metabolites identified based on variable importance in projection (VIP) scores. Positive and negative correlations are represented by varying intensities of red and green, respectively. Significantly correlated keystone taxa and metabolites are indicated on the heatmap. * *p* < 0.05, ** *p* < 0.01.

Spearman correlation analysis was conducted using 52 genera enriched in the CDS and SH groups, as well as the top 20 metabolites or six VFAs. Among the 1040 possible correlations (52 genera × 20 metabolites) in each group, 62 (positive: 19; negative: 43), 84 (48, 36), 160 (51, 109), and 100 (68, 32) significant correlations (*p* < 0.05) were observed in the CDS-H, CDS-D, SH-H, and SH-D groups, respectively, irrespective of correlation direction (Figure 4). Among the 312 possible correlations (52 genera × 6 VFAs), 29 (0, 29), 27 (3, 24), 115 (84, 31), and 36 (24, 12) significant correlations (*p* < 0.05) were observed in the CDS-H, CDS-D, SH-H, and SH-D groups, respectively (Figure 4). Notably, in the SH-H group, extensive correlations were observed between microorganisms and 2-hydroxy-3-methylbutyric acid or isobutyric acid, with 30 (7 positive and 23 negative) and 35 (31 positive and 4 negative) of the 52 genera showing significant correlations with these metabolites, respectively (Figure 4). These results suggest that microbiota–metabolite associations may be more prominent and structured in the SH group, particularly under non-diarrheal conditions.

## 4. Discussion

In Indonesia, the SH system remains the predominant calf-rearing practice, unlike most countries where CDS are more prevalent [32]. However, evidence on the impact of distinct farm management practices (i.e., SH and CDS) on the development of the calf gut environment in Indonesia remains insufficient. In our study, alpha diversity did not differ significantly among the four groups, consistent with previous reports showing that forage–concentrate differences often influence microbial composition without consistently affecting microbial richness in developing calves [6,33,34,35,36]. Moreover, our results showed that calf management systems influenced the microbial community structure and metabolic profiles without affecting the overall microbial richness [6,17,37]. Diarrhea in young calves is typically associated with reduced microbial richness and microbial community disruption, likely due to accelerated intestinal transit, mucosal damage, and overgrowth of opportunistic taxa [23]. However, in our study, diarrheal status did not alter microbial richness or community structure, suggesting that the disturbance primarily affected metabolic profiles associated with microbial function rather than the microbial composition [37]. It should be noted that diarrhea status is associated with reduced VFA absorption and a shift toward simple sugar fermentation, resulting in higher fecal VFA levels and altered metabolic pathways [37,38]. In addition, diarrhea interacts with diet, whereby post-weaning calves in the CDS experience a greater increase in fecal metabolites, such as VFAs, due to rapid starch fermentation and impaired absorption, whereas post-weaning calves in the SH system show a milder metabolic shift, as fiber fermentation is rapidly disrupted under reduced digesta retention time [6,14,38]. This pattern suggests that functional instability during diarrhea is more pronounced in concentrate-based systems, where rapid fermentation amplifies metabolic fluctuations. In contrast, the fiber-driven fermentation typical of SH calves may buffer against abrupt metabolic shifts, contributing to greater functional resilience despite similar microbial richness [17,39,40]. Therefore, although microbial richness remained stable even under diarrheal conditions, management structure in Indonesian calf-rearing systems primarily shapes metabolic resilience rather than taxonomic diversity, highlighting a decoupling between microbial composition and function.

In the microbe–metabolite correlation analysis, we observed distinct patterns between the Indonesian CDS and SH systems. Healthy post-weaning calves in the SH system showed a greater number of statistically significant correlations than both diarrheic calves in the SH system and calves in the CDS, irrespective of diarrhea status. These patterns suggest that microbe–metabolite associations differ between management systems, potentially reflecting contrasting fermentation environments created by concentrate-based versus forage-based feeding. High-starch diets are known to promote rapid fermentation, lower hindgut pH, and increase mucosal sensitivity [6,33,41]. The reduced number of correlations in diarrheic calves in the SH group may be related to shortened intestinal retention time during diarrhea, which limits microbial contact with substrates and reduces fiber fermentation [24,33,37,38]. Across both systems, healthy post-weaning calves exhibited predominantly negative correlations between the 52 genera and 20 metabolites, whereas diarrheic post-weaning calves showed a shift toward more positive associations. Post-weaning calves in the CDS also showed more correlations involving fecal VFAs than SH calves, with the strongest associations observed in diarrheic calves. These correlation patterns indicate that feeding management influences how microbial communities and metabolites covary, although these associations should be interpreted as statistical relationships rather than direct biological regulation.

Given the differences in feeding systems across Indonesian calf-rearing practices, our findings suggest several practical adjustments to strengthen gut health and improve performance in CDS and SH systems. Specifically, for the CDS, moderating the dietary starch load and incorporating small amounts of forage may help stabilize hindgut pH, reduce mucosal stress, and prevent excessive VFAs accumulation during diarrhea, as a forage-based diet is known to buffer rumen acidity and support more stable fermentation [6,14]. Increasing structural fiber can also enhance mucosal integrity and reduce the instability associated with rapid starch fermentation. For the SH system, improving forage quality and ensuring adequate nutrient density can support growth while maintaining the natural stability of fiber-driven fermentation [14]. Previous studies have shown that diarrhea accelerates transit, damages the mucosa, and disrupts fermentation efficiency [17,19]. Therefore, across both systems, management practices that protect mucosal health, such as gradual diet transitions, maintaining hygiene, and minimizing diarrhea triggers, can help improve fermentation stability and overall calf performance. Although the present study was not designed to define specific diarrhea-control strategies for each management system, our findings suggest that management-dependent differences in fermentation environments and metabolic resilience may provide an important framework for developing future system-specific approaches to calf health management.

## 5. Conclusions

Our findings suggest that differences between Indonesian CDS and SH systems are reflected more strongly in functional fermentation responses than in microbial richness. CDS post-weaning calves tended to show greater metabolic variability and fewer microbe–metabolite associations, particularly during diarrhea, whereas SH post-weaning calves maintained more stable, fiber-driven fermentation patterns. These observations are consistent with previous studies showing that high-starch diets promote rapid fermentation, lower hindgut pH, and increased mucosal sensitivity, while fiber-rich diets support more gradual fermentation and greater functional stability. From a practical perspective, moderating concentrate intake and increasing structural fiber, such as forage, may help reduce hindgut stress in CDSs, whereas improving forage quality and nutrient balance may further support gut function in SH systems. Therefore, aligning feeding strategies with the functional needs of the developing gut may enhance calf health and performance across Indonesian dairy production systems.

## Data Availability

All data involved in this study are available from the corresponding author upon request.

## Supplementary Materials

The following supporting information can be downloaded at: https://www.mdpi.com/article/10.3390/ani16121766/s1, Table S1: Results of phylum-level analysis based on 16S rRNA sequencing; Table S2: Results of family-level analysis based on 16S rRNA sequencing; Table S3: Results of genus-level analysis based on 16S rRNA sequencing; Table S4: Results of metabolomic analysis; Table S5: Results of VFA analysis; Table S6: Summary of metabolomic data quality assessment.

## Abbreviations

The following abbreviations are used in this manuscript:

CDS	Commercial Dairy System
SH	Smallholder farm
rRNA	Ribosomal Ribonucleic acid
GC-MS	Gas Chromatography–Mass Spectrometry
PCR	Polymerase Chain Reaction
OTU	Operational Taxonomic Unit
MSTFA	N-Methyl-N-trimethylsilyl-trifluoroacetamide
VFA	Volatile Fatty Acid
LDA	Linear Discriminant Analysis
PLS-DA	Partial Least-Squares-Discriminant Analysis
LEfSe	Linear Discriminant Analysis Effect Size
